# BTSD: A curated transformation of sentence dataset for text classification in Bangla language

**DOI:** 10.1016/j.dib.2023.109445

**Published:** 2023-07-24

**Authors:** Rajesh Kumar Das, Mirajul Islam, Sharun Akter Khushbu

**Affiliations:** Department of Computer Science and Engineering, Daffodil International University, Dhaka 1341, Bangladesh

**Keywords:** Natural language processing, Machine learning, Text classification, Transformation of sentence, Bangla language

## Abstract

The Bangla Transformation of Sentence Classification dataset addresses the resource gap in natural language processing (NLP) for the Bangla language by providing a curated resource for Bangla sentence classification. With 3,793 annotated sentences, the dataset focuses on categorizing Bangla sentences into Simple, Complex, and Compound classes. It serves as a benchmark for evaluating NLP models on Bangla sentence classification, promoting linguistic diversity and inclusive language models. Collected from publicly accessible Facebook pages, the dataset ensures balanced representation across the categories. Preprocessing steps, including anonymization and duplicate removal, were applied. Three native Bangla speakers independently assessed the Transformation of Sentence labels, enhancing the dataset's reliability. The dataset empowers researchers, practitioners, and developers to build accurate and robust NLP models tailored to the Bangla language. It offers insights into Bangla syntax and structure, benefiting linguistic research. The dataset can be used to train models, uncover patterns in Bangla language usage, and develop effective NLP applications across domains.

**Specifications Table**

**Table:** 

Subject	Computer Science
Specific subject area	Machine Learning, Natural Language Processing, Bangla Text Classification
Type of data	Text Files (xlsx-formatted)
How data were acquired	The data was collected from publicly open Facebook pages, Literature and news articles.
Data format	Raw and Filtered
Description of data collection	Data was extracted from different Facebook pages, literature, and news articles in Bangladesh. It was selectively collected to ensure distributed data for each data label. The dataset comprises a total of 3793 sentences with three distinct sentence types, including simple, compound, and complex sentences. Furthermore, the data has been annotated by native Bangla speakers.
Data source location	Publicly open Facebook pages
Data accessibility	Repository name: Mendeley Data Data identification number: 10.17632/4k964xyz65.2 Direct URL to data: https://data.mendeley.com/datasets/4k964xyz65/2

## Value of the Data

• The Bangla Transformation of Sentence Classification dataset fills a crucial gap in resources for the Bangla language in the field of natural language processing, specifically for sentence classification. It offers a carefully annotated and categorized dataset containing 3793 Bangla sentences, enabling the development and training of NLP models tailored to the unique characteristics of the Bangla language.
• The dataset's diverse representation of sentence types and source domains allows for advancements in understanding Bangla syntax and structure, making it a valuable resource for linguistic research.
• Researchers, practitioners, developers, and data scientists in the field of natural language processing can benefit from the Bangla Transformation of Sentence Classification dataset, as it provides valuable resources for building more accurate and robust NLP models tailored to the Bangla language. Linguists and language enthusiasts can leverage this dataset to gain insights into Bangla syntax and structure, promoting a better understanding of the language.
• The dataset can be used to train and evaluate NLP models for sentence classification in the Bangla language, leading to the development of more accurate and effective applications. Researchers can analyze the dataset to uncover patterns and trends in Bangla language usage across different domains, such as literature, news articles, and social media.

## 1 Objective

Bangla Transformation of Sentence Classification dataset is to provide a curated resource for NLP researchers and practitioners working on Bangla sentence classification. It aims to facilitate the development of tailored NLP models for the Bangla language by addressing the resource gap [1]. The dataset focuses on classifying Bangla sentences into three categories, promoting linguistic diversity and inclusive language models [2]. It serves as a benchmark for evaluating NLP model performance on Bangla sentence classification, enabling effective approach identification. The ultimate objective is to advance the understanding and processing of Bangla text, leading to more accurate and robust sentence classification models that benefit the Bangla-speaking population [3].

## 2 Data Description

**Fig. 1:**
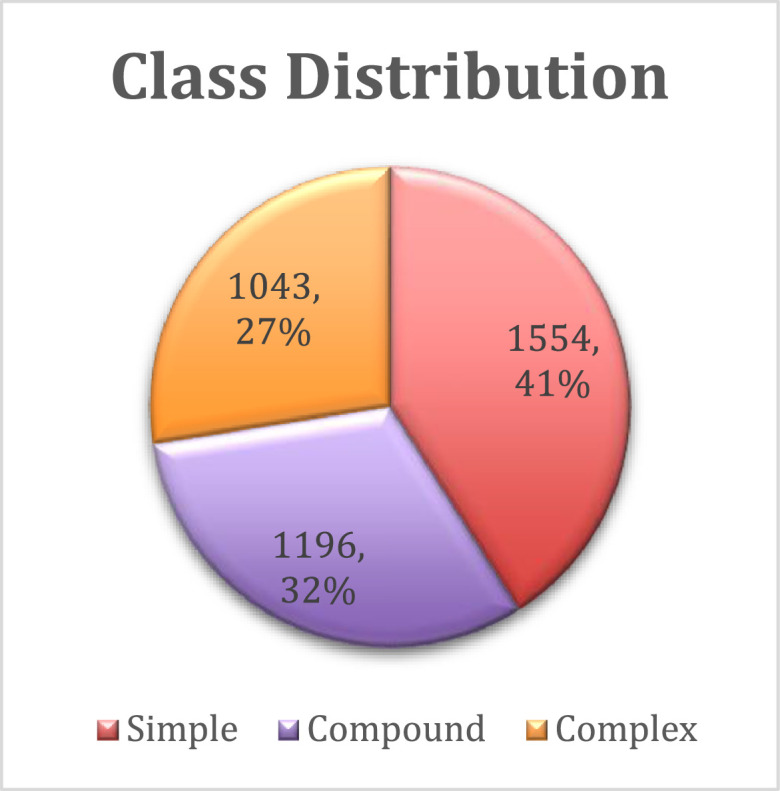
The class distribution of each label. (For interpretation of the references to color in this figure legend, the reader is referred to the web version of this article.)

The cornerstone of our research is the 'Bangla Transformation of Sentence Dataset (BTSD),' a meticulously curated collection of sentences specifically tailored for this study. The dataset, available as the raw data file named "Bangla Transformation of Sentence Dataset(BTSD).xlsx" in the repository, consists of 3793 sentences sourced from publicly accessible Facebook pages. The BTSD dataset has undergone careful curation to ensure its reliability and suitability for our research objectives. One crucial aspect of this curation process was maintaining an equal distribution of sentences across three distinct categories: Simple, Complex, and Compound. This balanced representation facilitates the model's ability to learn and generalize across various linguistic structures and complexities. Fig. 1 illustrates the distribution of sentence categories within the dataset. We acknowledge the significance of the Bengali language in our research context. Bengali belongs to the Indo-Aryan branch of the Indo-European language family, closely related to languages such as Assamese and Odia. It serves as the primary language in Bangladesh and the Indian states of West Bengal, Tripura, and Assam. Bengali is also spoken by diaspora communities worldwide. As the official language of Bangladesh and one of the 22 scheduled languages of India, Bengali boasts a substantial global speaker population, estimated at approximately 228 million [4]. Table 1 provides a detailed description of the variables present in the dataset.

**Table 1:** Dataset columns and its descriptions.

Variable name	Description
Raw Sentence In Bangla Language	The string representation of original text in the Bengali language. The original Bangla sentence obtained from Facebook pages.Example: সন্ধ্যায় পাখিরা বাসায় ফেরে (Birds return home in the evening) সন্ধ্যা হয় এবং পাখিরা বাসায় ফেরে (Dusk falls and the birds return home) যখন সন্ধ্যা হয় তখন পাখিরা বাসায় ফেরে (When the evening comes, the birds return home)
Labels of Transformation Sentence	The string representation of labels is assigned to each transformed sentence. The category of the sentence, classified as Simple, Complex, or Compound.Example: সরল বাক্য (Simple sentence) যৌগিক বাক্য (Compound sentence) জটিল বাক্য (Complex sentence)

**Fig. 2:**
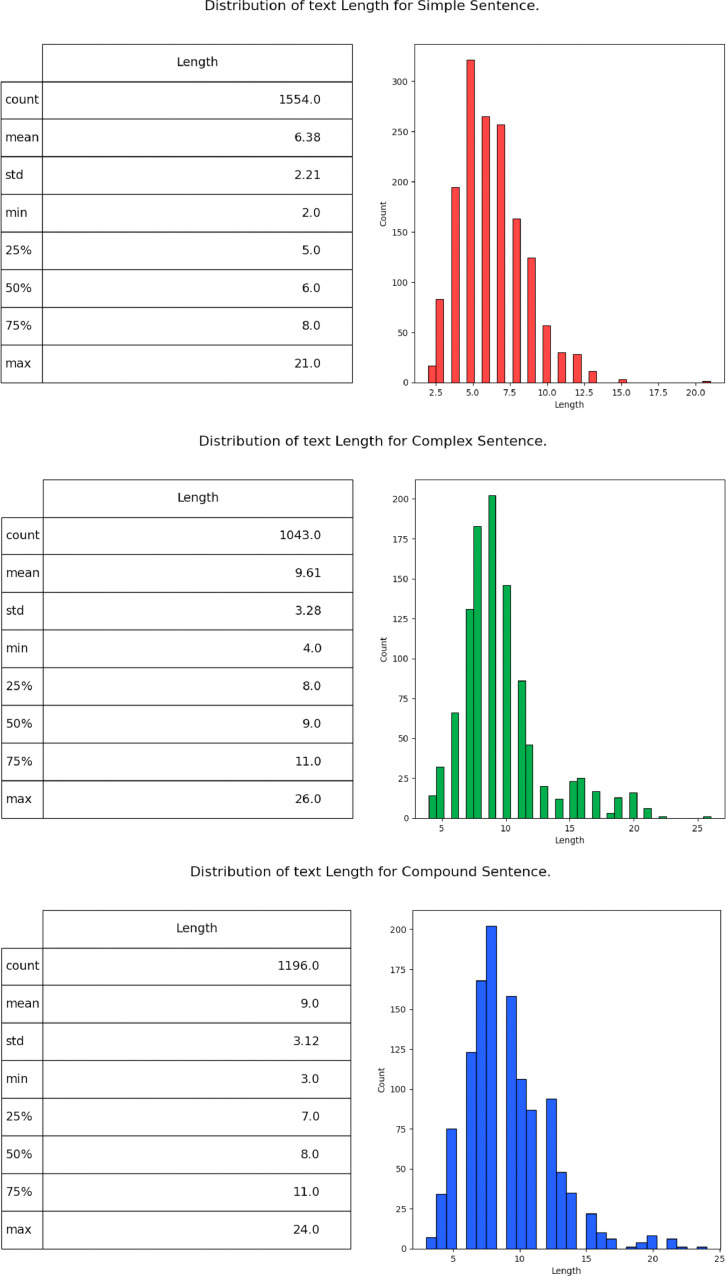
Distribution of text length (Simple, Complex, Compound). (For interpretation of the references to color in this figure legend, the reader is referred to the web version of this article.)

Fig. 2 depicts the distribution of text length within the dataset, specifically categorized into three types: Simple, Complex, and Compound. The graph provides insights into the varying lengths of sentences across these categories, highlighting potential differences in sentence structure and complexity. This information is crucial for developing a comprehensive dataset as it helps in understanding the distribution patterns and ensures a balanced representation of text lengths in the training data. It aids in creating models that can effectively handle sentences of different lengths, enhancing the dataset's usability for various natural language processing tasks.

Table 2 presents a list of the 20 most frequently occurring words in the dataset, along with their corresponding frequencies. However, it is important to acknowledge the limitations of this list. We did not remove stopwords from the dataset, which can impact the informativeness of the list. Stopwords are commonly used words in the language that do not carry significant meaning and are typically excluded from text analysis. Therefore, the inclusion of stopwords in the list may not provide a comprehensive representation of the most significant terms in the dataset. Nevertheless, analyzing the most common words still provides valuable insights into the common vocabulary present in the text samples. It helps identify significant linguistic features and patterns within the dataset, guiding the development of language models and algorithms. By focusing on the prevalent words, more accurate predictions and classifications can be achieved. Furthermore, the most common words list assists in data preprocessing tasks such as stop-word removal and feature selection, contributing to the creation of a more refined and effective dataset for training NLP models.

**Table 2:** Most 20 common words and it's frequency.

Common words	English translation	Frequency
তিনি	He/She	758
আমি	I	685
এবং	And	604
একটি	A/One	511
করতে	To do	485
না	No	476
সে	He/She	449
তারা	They	384
তার	His/Her	350
আমার	My	334
তখন	Then	305
যখন	When	301
তবে	However	263
করে	Doing	258
জন্য	For	247
আমরা	We	230
যদি	If	200
হবে	Will be	189
যেহেতু	Since	188
সেহেতু	Because	183

## 3 Experimental Design, Materials and Methods

The dataset creation workflow follows a systematic process. Initially, posts from Facebook were manually extracted, and their content was compiled into an Excel file. Subsequently, the aggregated dataset underwent several preprocessing steps, including anonymization, duplicate removal, and filtering out any instances of profanity language. In the third stage, a meticulous assessment of the dataset's Transformation of Sentence labels was carried out by three native Bangla speakers. Each assessor independently assigned labels based on three distinct polarities: Simple, Complex, and Compound.

The categorization of sentences into simple, complex, and compound is a widely recognized classification scheme employed in linguistic analysis to examine sentence structures across different languages, including Bengali. Although these classifications are not exclusive to Bengali linguistics, they serve as fundamental tools in the field of language analysis. To provide a more precise elucidation of these classifications [5]:

I. Simple Sentence: A simple sentence comprises a single independent clause that conveys a complete thought or idea. It consists of a subject and a predicate. For instance, the sentence "আমি বাংলা ভালবাসি।" (I love Bengali) exemplifies a simple sentence in Bengali.
II. Complex Sentence: A complex sentence encompasses an independent clause and one or more dependent clauses. Dependent clauses contribute supplementary information or contextual details to the independent clause. Consider the sentence "যখন আমি বাংলা পড়তে থাকি, আমি ভাল লাগে।" (When I study Bengali, I feel good). In this sentence, the dependent clause "যখন আমি বাংলা পড়তে থাকি" (When I study Bengali) provides additional information to the independent clause "আমি ভাল লাগে" (I feel good).
III. Compound Sentence: A compound sentence consists of two or more independent clauses connected by coordinating conjunctions or appropriate punctuation marks. Each independent clause can function independently as a separate sentence. For example, the sentence "আমি বাংলা পড়ি, এবং আমার বন্ধু বাংলা লিখে।" (I study Bengali, and my friend writes in Bengali) exemplifies a compound sentence. Here, the independent clauses "আমি বাংলা পড়ি" (I study Bengali) and "আমার বন্ধু বাংলা লিখে" (My friend writes in Bengali) are connected by the coordinating conjunction "এবং" (and).

The data was annotated by skilled native Bangla speakers following a comprehensive protocol: inter-annotator agreement (IAA) measures were employed. A subset of the data was randomly selected and annotated by multiple annotators independently. The annotations were then compared and analyzed for agreement using standard IAA metrics, such as Cohen's kappa coefficient or percentage agreement. The level of agreement between annotators was a crucial factor in ensuring the reliability and validity of the annotated dataset. Table 3 shows the annotation protocol methodological pseudo code.

**Table 3:** Annotation protocol.

Steps	|Description
1	| Start.
2	| Select skilled native Bangla speakers as annotators.
3	| Randomly select a subset of the data for annotation.
4	| Provide the selected subset to multiple annotators independently.
5	| Each annotator independently performs the annotation task following the guidelines.
6	| Compare and analyze the annotations for agreement.
7	| Calculate inter-annotator agreement using standard IAA metrics (e.g., Cohen's kappa coefficient, percentage agreement).
7.1	| Calculate Cohen's kappa coefficient:
	Ag = Proportion of observed agreement between annotators
	Eg = Proportion of expected agreement by chance
	kappa = (Ag - Eg) / (1 - Eg)
7.2	Calculate percentage agreement:
	Number of agreements = Count of annotations that match exactly between annotators
	Total number of annotations = Total count of annotations made by the annotators
	Percentage agreement = (Number of agreements / Total number of annotations) * 100
8	| Evaluate the level of agreement to ensure reliability and validity of the annotations.
9	| If agreement falls below the desired threshold, consider revising the guidelines or providing additional instructions.
10	| Iterate steps 3 to 9 until the desired level of agreement is achieved.
11	| End.

The accuracy of four state-of-the-art neural network-based deep learning models in classifying text data into three classes from our dataset was assessed. All models were trained for 50 epochs, where each epoch represents a complete pass through the entire dataset. The batch size was set to 64, indicating that the model would update its weights after processing 64 samples at a time. A comparative analysis was conducted to evaluate the performance of LSTM, bi-LSTM, Conv1D, and combined Conv1D-LSTM-based models, as outlined in Table 4. The highest accuracy of 91.17% was achieved by the Conv1D–LSTM Based Model.

**Table 4:** Performance of neural network-based deep learning models on our BTSD dataset.

Model name	Accuracy (%)	Class	Precision (%)	Recall (%)	F1 Score (%)
LSTM based model	91.04	Simple	90.20	91.54	90.86
		Complex	89.35	91.80	90.56
		Compound	93.15	90.07	91.58
Bi-LSTM based model	89.99	Simple	92.93	85.07	88.83
		Complex	87.45	92.58	89.94
		Compound	90.46	91.06	90.76
Conv1D based model	89.59	Simple	81.65	88.56	84.96
		Complex	91.25	93.75	92.49
		Compound	94.24	86.75	90.34
Conv1D–LSTM based model	91.17	Simple	89.90	93.03	91.44
		Complex	87.45	92.58	89.94
		Compound	95.71	88.74	92.10

This thorough assessment ensures the dataset's reliability and accuracy, enhancing its value for research purposes. The dataset presented in this article serves as a foundation for research not only in sentence classification but also opens avenues for exploration in various domains of language processing in the Bangla language. It provides a valuable resource for researchers seeking to delve into broader aspects of Bangla language analysis, contributing to advancements in the field of natural language processing and facilitating a deeper understanding of the intricacies of the Bangla language.

## Ethics Statements

No human or animal studies were conducted in this research. We anonymized all content from social media pages, and no records of personal information were kept. We adhered to Facebook's redistribution policies [6,7], and no permission was required for using content from publicly open Facebook pages.

## CRediT authorship contribution statement

**Rajesh Kumar Das:** Conceptualization, Visualization, Methodology, Data curation, Writing – original draft. **Mirajul Islam:** Investigation, Validation, Writing – review & editing. **Sharun Akter Khushbu:** Validation, Supervision.

## Data Availability

Bangla Transformation of Sentence Dataset (BTSD) (Original data) (Mendeley Data).

## References

[1] Karim M.A. (2013).

[2] Joshi, P., S. Santy, A. Budhiraja, K. Bali, and M. Choudhury. "The state and fate of linguistic diversity and inclusion in the NLP world." arXiv preprint arXiv:2004.09095 (2020).

[3] Sen O., Fuad M., Islam Md.N., Rabbi J., Masud M., Hasan Md.K., Awal Md.A. (2022). Bangla natural language processing: a comprehensive analysis of classical, machine learning, and deep learning based methods. IEEE Access.

[4] Sayeed A., Shin J., Hasan M.A.M., Srizon A.Y., Hasan M.M. (2021). BengaliNet: a low-cost novel convolutional neural network for Bengali handwritten characters recognition. Appl. Sci..

[5] Karim R. (2023). Simple, complex and compound sentence কাকে বলে?. Learn English with Rezaul.

[6] Facebook Team (2021). What is public information on Facebook?. Facebook.

[7] Facebook Team Page Public Content Access, Facebook, 2021 https://developers.facebook.com/docs/features-reference/page-public-content-access/. Accessed May 25, 2023.

